# Crustin Defense against *Vibrio parahaemolyticus* Infection by Regulating Intestinal Microbial Balance in *Litopenaeus vannamei*

**DOI:** 10.3390/md21020130

**Published:** 2023-02-17

**Authors:** Xinjia Lv, Shihao Li, Yang Yu, Xiaojun Zhang, Fuhua Li

**Affiliations:** 1 CAS and Shandong Province Key Laboratory of Experimental Marine Biology, Institute of Oceanology, Chinese Academy of Sciences, Qingdao 266071, China; 2 Laboratory for Marine Biology and Biotechnology, Qingdao National Laboratory for Marine Science and Technology, Qingdao 266237, China; 3 Center for Ocean Mega-Science, Chinese Academy of Sciences, Qingdao 266071, China; 4 The Innovation of Seed Design, Chinese Academy of Sciences, Wuhan 430072, China

**Keywords:** antimicrobial peptide, crustin, microbiota balance, *Vibrio parahaemolyticus*, *Litopenaeus vannamei*

## Abstract

Crustins are a kind of antimicrobial peptide (AMP) that exist in crustaceans. Some crustins do not have direct antimicrobial activity but exhibit in vivo defense functions against *Vibrio*. However, the underlying molecular mechanism is not clear. Here, the regulatory mechanism was partially revealed along with the characterization of the immune function of a type I crustin, *LvCrustin I*-2, from *Litopenaeus vannamei*. *LvCrustin I*-2 was mainly detected in hemocytes, intestines and gills and was apparently up-regulated after *Vibrio parahaemolyticus* infection. Although the recombinant LvCrustin I-2 protein possessed neither antibacterial activity nor agglutinating activity, the knockdown of *LvCrustin I*-2 accelerated the in vivo proliferation of *V. parahaemolyticus*. Microbiome analysis showed that the balance of intestinal microbiota was impaired after *LvCrustin I*-2 knockdown. Further transcriptome analysis showed that the intestinal epithelial barrier and immune function were impaired in shrimp after *LvCrustin I*-2 knockdown. After removing the intestinal bacteria via antibiotic treatment, the phenomenon of impaired intestinal epithelial barrier and immune function disappeared in shrimp after *LvCrustin I*-2 knockdown. This indicated that the impairment of the shrimp intestine after *LvCrustin I*-2 knockdown was caused by the dysbiosis of the intestinal microbiota. The present data suggest that crustins could resist pathogen infection through regulating the intestinal microbiota balance, which provides new insights into the functional mechanisms of antimicrobial peptides during pathogen infection.

## 1. Introduction

Antimicrobial peptides (AMPs) are a kind of immune effectors in innate immunity, which play essential functions in the immune defense of numerous organisms [1]. AMPs also present multiple functions including immune pathway activation, cytokine induction, endotoxin neutralization and microbiota modulation [2,3,4,5]. In invertebrates, due to the lack of adaptive immunity, the production of AMPs is an important strategy for defending against pathogens and regulating the host microbiota [6,7,8].

In crustaceans, different AMP families, including penaeidins, crustins, ALFs, etc., have been identified and characterized [9]. Crustins contain a whey acidic protein (WAP) domain and exhibit broad-spectrum activities against various microorganisms [10]. Since the first crustin gene was isolated from the hemocytes of *Carcinus maenas*, a variety of crustin genes have been identified in different crustaceans, including shrimp, crab, lobster, crayfish, etc. [11,12,13,14]. Crustins are classified into seven types of which type V is only found in insects and other types exist in crustaceans [15,16].

To date, the reported diverse types of crustins show different antimicrobial functions. Type I crustins show antimicrobial activity against Gram-positive bacteria, and weak activity against yeasts [9,17]. Type II, type III and type IV crustins show activities against both Gram-positive and Gram-negative bacteria [9,17,18]. Some crustins not only exhibit strong antimicrobial activities in vitro but also show immune defense function in vivo during pathogen infection [19,20].

Besides their direct antimicrobial activities against various microorganisms, other functions have been reported. A type III crustin from *L. vannamei* shows protease inhibition activity [21]. A type I crustin accumulates in damaged tissues in *C. maenas*, indicating a probable role in wound healing and tissue regeneration [22]. One type VII crustin from *L. vannamei* not only has antimicrobial activities against both Gram-positive and Gram-negative bacteria but also enhances hemocyte phagocytosis [23]. The expression of a type II crustin in *Rimicaris exoculata* spatiotemporally correlates with the establishment of ectosymbiotic microbiota [24]. The silencing of a gill-abundant type II crustin in *L. vannamei* results in a change in the proportion of the bacterial population in the gill microbiota [25].

Despite the multiple functions of crustins, knowledge about how they defend against pathogen infection in vivo is still limited. Previously, a type I crustin with an atypical WAP domain exhibited an intestinal microbiota-modulating function, and the knockdown of this gene accelerated the infection of *V. parahaemolyticus* [26]. However, the underlying molecular mechanism of how crustins affect the AHPND pathogenetic process is still unclear.

In the present study, we have characterized the function of a new type I crustin gene from *L. vannamei*, named *LvCrustin I*-2. The in vivo and in vitro activities and their effects on intestinal immune function and microbiota balance in shrimp were studied. The results suggest that *LvCrustin I*-2 plays important roles in maintaining the intestinal health of the shrimp by keeping the balance of microbiota, which will provide new clues for understanding the molecular mechanisms of AMPs modulating the outbreak of AHPND in shrimp aquaculture.

## 2. Results

### 2.1. LvCrustin I-2 Involved in Immune Defense during V. parahaemolyticus Infection

**Figure 1:**
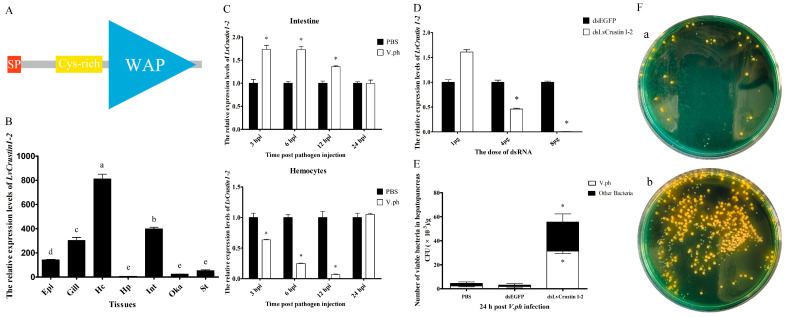
Characterization of the immune function of *LvCrustin I*-2 in shrimp during *V. parahaemolyticus* infection. (**A**) Schematic illustration of the primary structure of LvCrustin I-2. SP, Cys-rich and WAP presented the signal peptide, cysteine-rich region, and whey acidic protein domain, respectively. (**B**) Tissue distribution of *LvCrustin I*-2 transcripts. Epi, epidermis; Hc, hemocytes; Hp, hepatopancreas; Int, intestine; Oka, lymphoid organ; St, stomach. Vertical bars represented mean ± S.E. Letters “a”, “b”, “c”, “d” and “e” represented significant differences among treatments at *p* < 0.05. (**C**) Time-course expression pattern of *LvCrustin I*-2 after *V. parahaemolyticus* challenge in intestine and hemocytes: 3, 6, 12 and 24 hpi indicate 3 h, 6 h, 12 h and 24 h after pathogen injection. Significant differences between treatment and control groups are labeled with a star at *p* < 0.05. (**D**) Silencing efficiency of *LvCrustin I*-2 dsRNA in different dosages. The optimal silencing dose is marked with a star at *p* < 0.05. (**E**) The total viable bacteria count in hepatopancreas of *LvCrustin I*-2 silenced shrimp after *V. parahaemolyticus* infection. The data were obtained from three independent repeats with three individuals per sample. Significant differences between treatment and control groups are labeled with a star at *p* < 0.05. (**F**) Spread plates of hepatopancreas homogenate of shrimp infected with *V. parahaemolyticus* after dsEGFP injection (a) or dsLvCrustin I-2 injection (b).

The cDNA sequence of *LvCrustin I*-2 (accession number: MT375558.1) contained a 345 bp open reading frame encoding 114 deduced amino acid (aa) residues (Figure S1). According to the protein sequence, which contains a 19-aa signal peptide, a cysteine-rich region (Cys^31^~Cys^45^) and a 51-aa whey acidic protein (WAP) domain (Lys^58^-Ser^108^), LvCrustin I-2 was classified as a type I crustin (Figure 1A). Tissue distribution analysis showed that *LvCrustin I*-2 had the highest expression level in hemocytes, followed by the intestine, gill and epidermis and a relatively low expression level in the stomach, Oka and hepatopancreas (Figure 1B).

The time-course expression pattern of *LvCrustin I*-2 was analyzed in the hemocytes and intestine of shrimp after *V. parahaemolyticus* challenge. The expression level of *LvCrustin I*-2 was up-regulated by 1.73-fold, 1.73-fold and 1.35-fold in intestine at 3, 6 and 12 hpi, respectively. However, it decreased to 0.64-fold, 0.25-fold and 0.07-fold in hemocytes at 3, 6 and 12 hpi (Figure 1C).

RNAi was used to investigate the immune function of *LvCrustin I*-2 during *V. parahaemolyticus* infection. The interference efficiency of *LvCrustin I*-2 was 54% and 99.16% at the dosages of 4 μg and 8 μg dsRNA per shrimp, respectively (Figure 1D). Therefore, the dosage of 8 μg dsRNA per shrimp was used to study the immune function of *LvCrustin I*-2. The number of bacteria in the hepatopancreas could reflect the health status of shrimp. To study the impact of *LvCrustin I-*2 silencing on the *V. parahaemolyticus* infection process, the amount of *Vibrio* in the hepatopancreas of *LvCrustin I-*2-silenced shrimp was detected. After *LvCrustin I*-2 knockdown, the amount of total viable *V. parahaemolyticus* in hepatopancreas was 3.15 × 10^4^ cfu/g, which was significantly higher than those from the PBS group (2.34 × 10^3^ cfu/g) and dsEGFP group (1.82 × 10^3^ cfu/g) (Figure 1E). In addition, the amount of other viable bacteria, including *V. harveyi* and *P. damselae*, was also higher in *LvCrustin I*-2-silenced shrimp (2.41 × 10^4^ cfu/g) than those from the PBS group (2.11 × 10^3^ cfu/g) and dsEGFP group (1.36 × 10^3^ cfu/g) (Figure 1E).

To further study the immune function of *LvCrustin I-2*, in vitro activities were analyzed. The recombinant protein (rLvCrustin I-2) was expressed in *E. coli* with a predicted molecular mass of 28.53 kDa (Figure S2, lanes 1 and 2). rLvCrustin I-2 was mainly produced in a soluble form (Figure S2, lines 3 and 4) and then purified (Figure S2, lane 5). The MIC assay and microorganism-binding assay showed that rLvCrustin I-2 had no antibacterial activity (Table S1) or binding activity (Figure S2) on pathogens, including *V. parahaemolyticus*, *V. harveyi* and *P. damselae*. A further agglutination test showed that rLvCrustin I-2 had no agglutinating activity to *V. parahaemolyticus* (Figure S3).

### 2.2. Imbalance of the Intestinal Microbiota after LvCrustin I-2 Knockdown

The microbiota between the PBS group and dsEGFP group were first compared. The alpha diversity analysis showed there was no significant difference in all the alpha indexes including Ace, Chao1, Simpson and Shannon through Welch’s *t*-test (*p*-values were 0.92, 0.62, 0.71 and 0.96, respectively) or by Wilcoxon rank test (*p*-values were 1, 0.7, 0.7 and 0.7, respectively). The beta diversity analysis showed that the inter-group difference between the PBS group and dsEGFP group was less than the intra-group difference, as compared by the ANOSIM test (*p*-value was 0.2) and ADONIS test (*p*-value was 0.4). To facilitate the subsequent analysis, we combined the samples from the PBS group and dsEGFP group into the “control group”.

**Figure 2:**
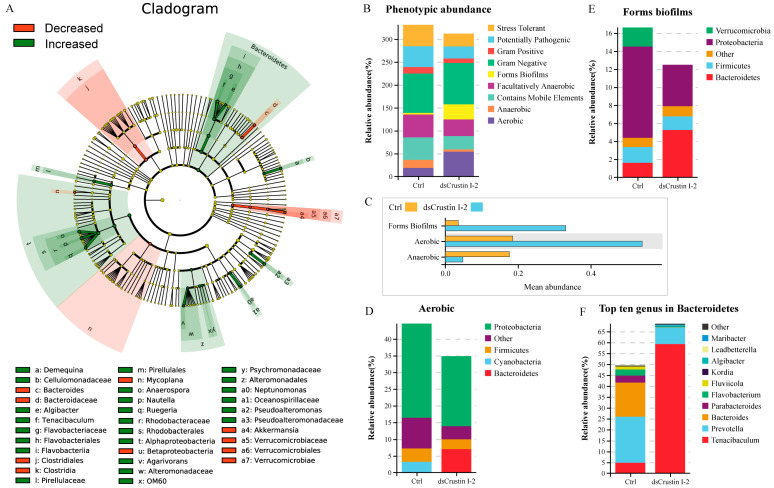
Intestinal microbiota analysis in shrimp after *LvCrustin I*-2 knockdown. (**A**) LEFse analysis of the intestinal microbiota of the control group and dsLvCrustin I-2 group. The green and red colors indicate increased and decreased bacteria in different taxa, respectively. The “Decreased” indicate the bacteria have higher proportion in the control group than that in the dsLvCrustin I-2 group. The “Increased” indicate the bacteria have higher proportion in the dsLvCrustin I-2 group than that in the control group. (**B**) Predicted functional profile of intestinal microbial community was classified into nine phenotypes. (**C**) Mean abundance of phenotypes at *p* < 0.05. (**D**) Relative abundance of different phyla in biofilm-forming phenotype. (**E**) Relative abundance of different phyla in aerobic phenotype. (**F**) Top ten genera in Bacteroidetes.

The alpha diversity analysis showed that the Ace index increased from 785.58 in the control group to 876.86 in the dsLvCrustin I-2 group (Table S2). However, the Chao1 index, Simpson index and Shannon index had no significant change (Table S2). The result of principal co-ordinate analysis (PCoA) based on weighted UniFrac distance showed that the samples in the dsLvCrustin I-2 group and the control group could be obviously divided into two clusters through ADONIS analysis (Figure S4, *p*-value is 0.023). After *LvCrustin I*-2 knockdown, the proportion of bacteria at different taxonomic levels significantly changed in the intestinal microbiota of shrimp (Figure 2A and Figure S5). Among the top ten genera, the proportion of *Tenacibaculum*, *Anaerospora*, *Nautella* and *Pseudoalteromonas* increased from 0.30%, 0.12%, 0.39% and 0.22% to 9.11%, 2.9%, 1.83% and 4.23%, respectively (Figure S5E and Figure 2A, f, o, p and a2), followed by *Demequina*, *Algibacter*, *Ruegeria*, *Agarivorans* and *Neptunomonas* (Figure 2A, a, e, q, v and a0). However, the proportion of *Bacteroides*, *Mycoplana* and *Akkermansia* significantly decreased (Figure 2A, c, n and a4).

The functional profile of the intestinal microbial community was predicted. The intestinal microbiota was classified into nine phenotypes. The proportions of biofilm-forming and aerobic bacteria were significantly higher in the dsLvCrustin I-2 group (Figure 2B,C), which was attributed to the abundance of Bacteroidetes (Figure 2D,E). Whereas the proportion of anaerobic bacteria was significantly higher in the control group (Figure 2B,C). Therefore, we further analyzed the bacteria in Bacteroidetes. Among the top ten genera in Bacteroidetes, *Tenacibaculum* and *Algibacter* were also the biomarkers of the dsLvCrustin I-2 group (Figure 2A, f and e). The proportions of *Tenacibaculum* and *Algibacter* in Bacteroidetes increased from 4.83% and 0.03% to 59.26% and 0.50%, respectively, after LvCrustin I-2 knockdown (Figure 2F).

### 2.3. Dysfunction of the Intestinal Epithelium after LvCrustin I-2 Knockdown

**Figure 3:**
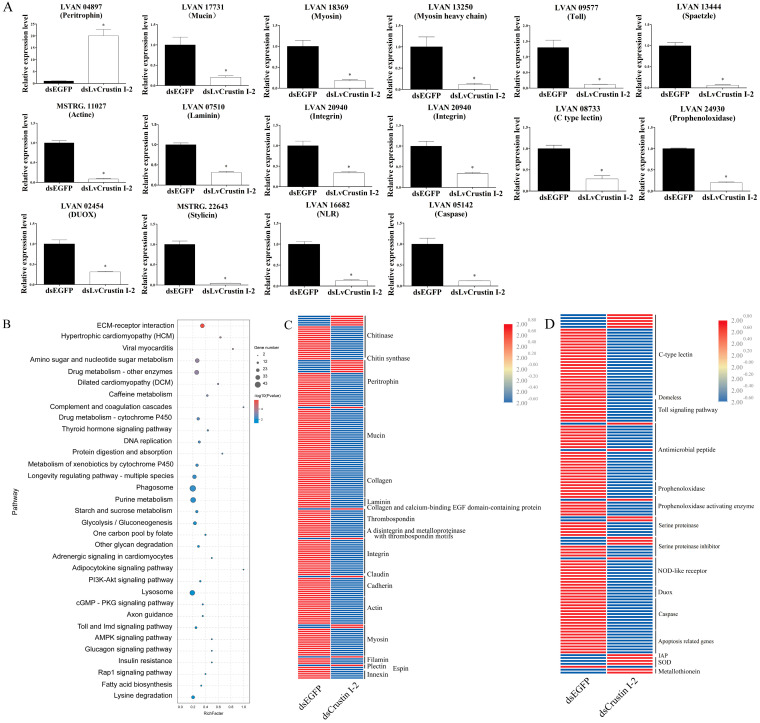
(**A**) Real-time quantitative PCR (qRT-PCR) analysis of candidate genes. The expression levels of these genes are presented as mean ± S.D. The gene 18S rRNA was used as the reference gene. The expression levels are all obtained from three biological replicates. Star (*) indicates statistical difference between dsLvCrustin I-2 group and dsEGFP group (One-way ANOVA, *p* < 0.05). The accession numbers of these selected genes are shown in Tables S3 and S4. (**B**) KEGG pathway enrichment analysis. “GeneNumber” represents the number of DEGs enriched in certain pathways. “*p* value” is the value obtained via hypergeometric test to define the significance of enriched pathways, and its colors represent the significance from low (blue) to high (red). (**C**) The DEGs involved in intestinal epithelial barrier integrity. (**D**) The DEGs involved in immune and oxidative stress. The colors represent the relative expression levels from low (blue) to high (red).

In order to further investigate the functional mechanism of *LvCrustin I*-2, the intestinal transcriptomes of shrimp before and after *LvCrustin I*-2 knockdown were compared. Detailed information on the sequencing and assembly of the intestinal transcriptome is shown in Table S3. A total of 3329 DEGs were obtained, including 2396 differentially down-regulated genes (DDGs) and 933 differentially up-regulated genes (DUGs) in the dsLvCrustin I-2 group. QPCR analysis of 16 selected DEGs showed that they were all consistent with the transcriptome data (Figure 3A).

KEGG analysis showed that many enriched pathways were associated with cell and extracellular matrix interactions, including “ECM-receptor interaction”, “Hypertrophic cardiomyopathy (HCM)”, “Viral myocarditis” and “Dilated cardiomyopathy (DCM)” (Figure 3B). The genes in these pathways, including *integrin*, *actin*, *myosin* and many components of the extracellular matrix, were associated with the cell morphology and structural integrity of the intestinal epithelial barrier. Pathways including the “Toll and Imd signaling pathway”, “Phagosome” and “Lysosome” were associated with the immune and oxidized stress responses of shrimp. KEGG analysis also identified many DEGs related to intestinal epithelial barrier integrity, immunity and the oxidized stress response (Tables S4 and S5). Most of the DEGs involved in cell junction and adhesion and enterocyte microvilli structure were significantly down-regulated in the dsLvCrustin I-2 group (Figure 3C). Most of the DEGs involved in immune responses and cell apoptosis were also significantly down-regulated in the dsLvCrustin I-2 group, while the apoptosis-inhibiting related genes and antioxidant genes were significantly up-regulated (Figure 3D).

### 2.4. Epithelial Function Was Not Impaired in LvCrustin I-2-Silenced Shrimp after Removing Intestinal Microbiota

**Figure 4:**
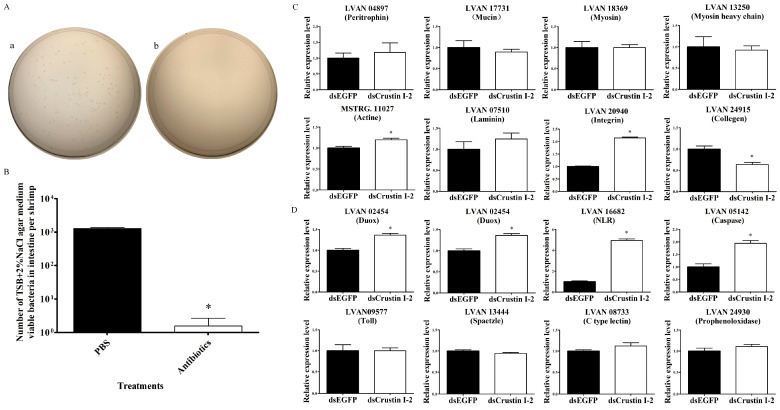
Detection of intestinal epithelial barrier and immune-related genes in shrimp after removing intestinal microorganisms and *LvCrustin I-2* knockdown. (**A**) Spread plates of intestine homogenate of shrimp. (a) Intestine homogenate of PBS buffer-treated shrimp. (b) Intestine homogenate of antibiotic-treated shrimp. (**B**) Total viable bacteria counts in intestines of shrimp treated with PBS buffer or antibiotics. The data were obtained from three independent repeats with three individuals per sample. Significant differences between treatment and control groups are labeled with a star at *p* < 0.05. (**C**) qRT-PCR analysis of intestinal epithelial barrier-related genes. (**D**) qRT-PCR analysis of immune-related genes. The expression levels of these genes are presented as mean ± S.D. The gene 18S rRNA was used as the reference gene. The expression levels were all obtained from three biological replicates. Star (*) indicates statistical difference between the dsLvCrustin I-2 group and dsEGFP group (One-way ANOVA, *p* < 0.05).

In order to find out the causal relationship between the epithelial functional impairment and microbiota imbalance in the intestine after *LvCrustin I*-2 knockdown, expression profiles of DEGs related to intestinal function were tested in shrimp after removing intestinal microorganisms. At 48 h after the last reverse perfusion, the TSB+2%NaCl agar medium viable bacteria dramatically decreased from 7.66 × 10^6^ cfu/g (1275.56 bacteria per shrimp) to 9.33 × 10^3^ cfu/g (1.56 bacteria per shrimp) (Figure 4A,B). After removing intestinal microorganisms, *LvCrustin I*-2 knockdown did not affect the expression of most of the intestinal epithelial barrier-related genes, including peritrophin, mucin, myosin, myosin heavy chain and laminin (Figure 4C). Some immune-related genes, including Duox, stylicine, NLR and caspase, were even upregulated after *LvCrustin I*-2 knockdown (Figure 4D).

## 3. Discussion

Many crustacean AMPs have been reported to regulate pathogen infection through direct antimicrobial activity. The knockdown of AMPs with antibacterial activity against the pathogens led to an increase in bacterial count in tissues or a higher mortality rate after infection [27,28,29]. In the present study, the knockdown of *LvCrustin I-*2 accelerated the propagation of *V. parahaemolyticus* and other viable bacteria in the hepatopancreas of infected shrimp. However, *LvCrustin I-*2 did not show antibacterial activity or agglutinating activity against the pathogenic bacteria *V. parahaemolyticus*. Therefore, the in vivo inhibition of *LvCrustin I-*2 on *V. parahaemolyticus* propagation might be attributed to its influence on the host defense and microbial balance of target tissues.

In addition to direct antimicrobial activity, AMPs also play a major role in regulating the balance between homeostasis and pathogenesis in crustaceans [9]. The interaction between internal microorganisms and the host immune system is essential to the health and survival of crustaceans [30]. The silencing of AMP genes led to an increase in the internal bacteria in tissues and resulted in crustacean death [31,32]. The expression of AMPs affects not only the amount but also the composition of bacteria in tissues of crustaceans [25]. In the present study, the intestinal microbiota composition significantly changed in shrimp after *LvCrustin I-*2 knockdown. The proportion of potentially pathogenic bacteria, including *Tenacibaculum*, *Nautella* and *Demequina* [33,34,35,36], significantly increased in shrimp after *LvCrustin I-*2 knockdown.

Stability and diversity are essential to a healthy intestinal microbial community [37]. A change in composition may affect the function of the microbial community. The proportion of strictly anaerobic bacteria and aerobe bacteria is an indicator of healthy intestinal microbiota [38]. The overgrowth of aerobe bacteria is independently linked with intestinal diseases [39]. In addition, the formation of biofilm facilitates pathogens’ resistance against the host immune system [40]. In our results of functional prediction, the high abundance of biofilm-forming and aerobic bacteria in shrimp after *LvCrustin I-*2 knockdown was attributed to the abundance of Bacteroidetes. Among the top ten genera in Bacteroidetes, the proportion of *Tenacibaculum* in the *LvCrustin I-*2 knockdown group was significantly higher than that in the control group. *Tenacibaculum* is a kind of pathogen that infects various marine organisms [33,41]. To date, there is no direct evidence showing that *Tenacibaculum* is a kind of shrimp pathogen. However, in the intestine of “cotton shrimp-like” diseased shrimp, the *Tenacibaculum* levels significantly increased, along with the down-regulated expression of immune genes [42]. In addition, many recent studies have shown that the supplementation of probiotics decreases the proportion of *Tenacibaculum* in the microbiota and improves intestinal health, the immune response and resistance to *Vibrio parahaemolyticus* infection [43,44,45]. Therefore, we considered *Tenacibaculum* to have an important role in microbiota dysbiosis and to be an indicator of the intestinal health of shrimp.

Intestinal dysbiosis can result in adverse effects on the host’s health [46]. The controlled production of reactive oxygen species (ROS) is an important way for regulating intestinal microorganisms and the induction of epithelial renewal and immune response in invertebrates [47,48]. However, an excessive ROS level could be deleterious and trigger oxidative stress, which damages the epithelial barrier along with pathogenic bacteria invasion [49]. In the present study, the up-regulated expression of antioxidant enzymes such as superoxide dismutase (SOD) and metallothionein implied that more ROS was probably produced in the intestine of shrimp after *LvCrustin I-*2 knockdown. It was reported that the high ROS level could inhibit the expression of Duox in shrimp [50]. The down-regulation of Duox and apoptosis-related genes as well as the up-regulation of inhibitors of apoptosis (IAP) indicated the suppression of ROS production and apoptosis of intestinal epithelial cells. Consequently, we speculated that the suppression of ROS production and apoptosis caused by the negative regulation of organisms was due to the excessive ROS level.

The integrity of the intestinal barrier, which includes the basal lamina, intestinal epithelium, mucus and peritrophic matrix, is important for maintaining the functions of the intestine in invertebrates [51]. After *LvCrustin I-*2 knockdown, genes involved in the intestinal epithelial barrier and immune response were down-regulated, indicating that the integrity and function of the intestinal barrier were impaired. Pathogens could disrupt or cross the barriers and cause infection by interacting with and manipulating components of epithelial barriers [52]. The impairment of the epithelial barrier and immune function may facilitate *V. parahaemolyticus* crossing the intestinal epithelium of shrimp and accelerate the infection progress.

As one kind of immune effector, crustins are the downstream molecules of immune signaling pathways. It is interesting to know why the knockdown of a single downstream gene could cause such a high level of gene expression changes. According to the above analysis, we proposed a hypothesis that the knockdown of *LvCrustin I-*2 influenced the intestinal microorganisms, and then the rupture of gut homeostasis by the microorganisms induced excessive ROS production and damaged the integrity of the intestinal barrier. In order to prove the hypothesis, the *LvCrustin I-*2 RNAi assay was performed in antibiotic-treated shrimp. After intestinal bacteria were removed, the knockdown of *LvCrustin I-*2 did not lead to a wide down-regulation of the intestinal epithelial barrier or immune-related genes, indicating the impairment of the epithelial barrier and immune function was directly caused by the dysbiotic microbiota.

In humans, intestinal dysbiosis is associated with host health and raises the risk of developing diseases [53,54]. In aquatic invertebrates, dysbiosis also leads to diseases. Dysbiosis was detected in the sea cucumber with skin ulceration syndrome due to a decrease in microbiota diversity [55]. The dysbiosis in the host intestinal microbiota played the causative role in the occurrence of shrimp white feces syndrome [56]. A study showed dysbiotic microbiota associated with an early development of AHPND [57]. To date, studies on the pathogenesis of AHPND have made progress [58,59]. However, it is still largely unknown how *V. parahaemolyticus* and the toxin migrated into the hepatopancreas. Many researchers have proposed hypotheses in response to this question [60]. A comprehensive model that incorporated dysbiotic microbiota was proposed by Kumar [61]. In this model, the entry of *V. parahaemolyticus* leads to dysbiosis of the microbiota, which enables the pathogen to further replicate and colonize, which in turn causes inflammation and an increased immune response. At the same time, the Rho pathway is activated by an unknown mechanism, which causes disruption of the cell junctions and disintegrates the epithelial barrier. In the present study, the dysbiotic microbiota impaired epithelial barrier function, which provided experimental evidence for the “unknown mechanism” in Kumar’s model.

## 4. Materials and Methods

### 4.1. Animals and Tissue Collection

Experimental shrimp *L. vannamei* with an average body length of 6.56 ± 0.54 cm and body weight of 3.42 ± 0.70 g obtained from Xingguang Marine Ranch Fishery Co., Ltd (Rizhao, Shandong, China). were cultured in our aquarium with aerated seawater at 25 °C and 27‰ salinity and fed with sterile commercial feed (DaLe, Yantai, Shandong, China) more than two months before being used. To paralyze and kill the shrimp, the nerves were cut off and the cephalothoraxes were removed. Tissues including lymphoid organ (Oka), hepatopancreas, intestine, stomach, gills, and epidermis from 12 shrimp were collected. Hemolymph was drawn and centrifuged at 800× *g*, 4 °C for 10 min to collect hemocytes. Each kind of tissue contained three samples, and each sample included the same tissues from 15 individuals. All the samples were stored at −80 °C for detection of the expression levels of the target genes.

### 4.2. Total RNA Extraction and RT-qPCR Analysis

Total RNA from 100 mg tissue sample was extracted using RNAiso Plus reagent (TaKaRa, kusatsu, Shiga, Japan). The concentration and quality of RNA samples were assessed using Nanodrop 2000 (Thermo Fisher Scientific, Waltham, MA, USA) and electrophoresis on 1% agarose gel. The first-strand cDNA was synthesized using PrimeScript™ RT reagent Kit with gDNA Eraser (TaKaRa, kusatsu, Shiga, Japan).

Quantitative real-time PCR (qPCR) was performed to detect the expression levels of *LvCrustin I-2* in different samples with primers qLvCrustin I-2-F and qLvCrustin I-2-R (Table S6). A pair of primers, 18S-F/R (Table S6), was designed to amplify 18S rRNA, which was used as an internal reference gene. The program of qPCR was set as follows: 95 °C for 1 min, followed by 40 cycles of 95 °C for 15 s, at the annealing temperature for 15 s, 72 °C for 30 s, and a melting curve analysis was used to verify the specificity of the product. The data were processed using 2^−ΔΔCT^ method [62].

### 4.3. Gene Cloning and Sequence Analysis

The cDNA sequence of *LvCrustin I-*2 was obtained from a transcriptome database of *L. vannamei* [63]. Primers *LvCrustin I-*2-F and *LvCrustin I-*2-R were synthesized to amplify the open reading frame (ORF) sequence of *LvCrustin I-*2. The PCR program was as follows: 95 °C for 5 min, followed by 35 cycles of 95 °C for 30 s, 55 °C for 30 s, 72 °C for 30 s, followed by an extension at 72 °C for 10 min. The PCR product was purified using MiniBEST DNA Fragment Purification Kit (TaKaRa, kusatsu, Shiga, Japan) and sub-cloned into the pMD19-T vector (TaKaRa, kusatsu, Shiga, Japan). The ORF sequence of the target gene was confirmed through Sanger sequencing with universal primers RV-M and M13-47 (Table S6).

The nucleotide sequence and deduced amino acid sequence of *LvCrustin I-*2 were analyzed using BLAST algorithm (NCBI, blast.ncbi.nlm.nih.gov/Blast.cgi, accessed on 15 March 2022). The signal peptide was predicted via CBS prediction servers (http://services.healthtech.dtu.dk/service.php?SignalP-5.0, accessed on 15 March 2022). The WAP domain was predicted with the InterPro servers (http://www.ebi.ac.uk/interpro/, accessed on 15 March 2022). Multiple sequence alignment was calculated using DNAMAN software (Version 7.0).

### 4.4. Pathogen Challenge and Gene Expression Analysis

The cDNA samples of *V. parahaemolyticus-*challenged shrimp were obtained as previously described [26]. Briefly, each shrimp in bacterial challenge group was injected with 2 × 10^5^ CFU *V. parahaemolyticus*. The shrimp in the control group were injected with equal volume of PBS buffer. The hemocytes and intestines were collected at 3 h, 6 h, 12 h and 24 h post-injection (hpi). Each time point contained three repeats of 15 shrimp. The total RNA of each sample was extracted, and the cDNA was synthesized as described in section “Total RNA Extraction and RT-qPCR Analysis”. The expression levels of *LvCrustin I-*2 in different samples were detected via qRT-PCR as described in section “Total RNA Extraction and RT-qPCR Analysis”.

### 4.5. Total Viable Bacteria Count after DsRNA and V. parahaemolyticus Injection

Primers dsLvCrustin I-2-F and dsLvCrustin I-2-R (Table S6) were designed to amplify the DNA template for *LvCrustin* I-2 dsRNA synthesis. The PCR was performed using the ExTaq (TaKaRa, Japan) with the program set as: 95 °C for 5 min, followed by 35 cycles of 95 °C for 30 s, 60 °C for 30 s, 72 °C for 40 s, followed by an extension at 72 °C for 10 min. The PCR product was purified with the Gel Extraction Kit (OMEGA, Norcross, GA, USA). The *LvCrustin I*-2 dsRNA was synthesized with the TranscriptAid T7 High Yield Transcription Kit (Thermo Fisher Scientific, Waltham, MA, USA) and purified with phenol–chloroform solution. The dsRNA of a 289 bp fragment of enhanced green fluorescent protein (EGFP) gene was also synthesized as negative control. The template for EGFP dsRNA was amplified from the pEGFP-N1 plasmid with a pair of primers, dsEGFP-F/R (Table S6). To optimize the interference doses, 30 individuals with a body weight of 4.75 ± 0.92 g were equally divided into two groups: dsLvCrustin I-2 group and dsEGFP group. Each group contained three dosages, including 1 μg, 4 μg and 8 μg dsRNA per shrimp. The intestines of five shrimp in each subgroup were collected as one sample 48 h after dsRNA interference. Total RNA extraction and qRT-PCR analysis were performed according to section “Total RNA Extraction and RT-qPCR Analysis”.

After dose optimization, 60 shrimp were equally divided into three groups: the PBS, dsEGFP, and dsLvCrustin I-2 groups. The shrimp in dsLvCrustin I-2 group and dsEGFP group were injected with 8 µg corresponding dsRNA per shrimp, respectively. The shrimp in PBS group were injected with equal volume of PBS buffer. At 48 h after dsRNA interference, the shrimp were injected with 2 × 10^4^ cfu *V. parahaemolyticus*. The hepatopancreas from three individuals was collected and crushed in sterile PBS buffer as one sample 24 h after bacterial infection. Each group contained three biological replicates. The tissue homogenate was seeded onto the TCBS (LuQiao, Beijing, China) agar medium and cultured at 28 °C overnight. The total viable bacteria were counted, and the dominant bacteria were identified with bacterial 16S rDNA sequencing method.

### 4.6. Recombinant Expression and Purification of rLvCrustin I-2

The vector pET32a, digested by restriction enzymes *Nco* I and *EcoR* I (TaKaRa, kusatsu, Shiga, Japan), was used to construct the recombinant protein expression plasmid. The DNA fragment encoding LvCrustin I-2 mature protein was amplified from the plasmid that contained the ORF sequence of target gene with primers rLvCrustin I-2-F/R (Table S6). The linearized vector and the DNA fragment were purified and linked using MiniBEST DNA Fragment Purification Kit (TaKaRa, kusatsu, Shiga, Japan) and In-Fusion HD Cloning Kit (TaKaRa, kusatsu, Shiga, Japan) according to the protocol from the manufacturer. The plasmid was transformed into TransB Competent Cell (TransGen, Beijing, China). The soluble rLvCrustin I-2 was induced to express by addition of 1 mM Isopropyl-b-d-thio-galactoside (IPTG) at 37 °C for 4 h and purified using HisTALON Gravity Column Purification Kit (Clontech, Mountain View, CA, USA). The tag protein of pET32a, thioredoxin (Trx), was also expressed and purified as a control. Purity of rLvCrustin I-2 and rTrx was verified using sodium dodecyl sulfate–polyacrylamide gel electrophoresis (SDS-PAGE) and visualized with Coomassie brilliant blue R250. The concentration of recombinant proteins was detected with the BCA Protein Quantification Kit (Vazyme, Nanjing, Jiangsu, China).

### 4.7. Minimal Inhibitory Concentration (MIC) Assay

The concentration of imidazole in protein solution (50 mM sodium phosphate, 300 mM sodium chloride and 150 mM imidazole) was diluted 300-fold using a centrifugal filter device (Millipore, Cork, Ireland) by adding desalting buffer (50 mM sodium phosphate, 300 mM sodium chloride) and centrifuging. The concentration of recombinant proteins was also adjusted to 1 mg/mL with the centrifugal filter device.

Bacterium strains including *V. parahaemolyticus*, *Vibrio harveyi*, *Photobacterium damselae* and *Escherichia coli* were cultured to logarithmic phase and counted on a blood cell counting plate under a microscope. The recombinant proteins were diluted to concentrations of 1 mg/mL, 0.500 mg/mL, 0.250 mg/mL, 0.125 mg/mL, 0.0625 mg/mL and 0.0313 mg/mL. A total of 50 μL of recombinant proteins with density gradient was incubated with 50 μL of 5 × 10^3^ cfu/mL bacterial suspension of each strain for 2 h in the 96-well plates at room temperature. After incubation, 150 μL TSB + 2% NaCl or LB liquid medium was added, and the plates were incubated at 28 °C or 37 °C for 8 h depending on different bacterial strains. The absorbance at 560 nm was detected via the precision micro-plate reader (TECAN infinite M200 PRO, Salzburg, Austria). The experiment was performed in triplicate.

### 4.8. Microorganism-Binding Assay

The microorganism-binding activity of rLvCrustin I-2 was detected through Western blot assay. The bacteria including *V. parahaemolyticus*, *V. harveyi*, *P. damselae* and *E. coli* were cultured to logarithmic phase. The number of each bacterial strain was counted using a blood cell counting plate under a microscope. About 1 × 10^8^ cfu of each kind of bacteria was washed with phosphate buffer saline (PBS) (Sangon, Shanghai, China) and then resuspended with 450 μL PBS. A total of 50 μL of rLvCrustin I-2 or rTrx was added to the bacterial suspension, with a final concentration of 200 μg/mL. One hour after incubation with the recombinant protein, the bacterial suspension was centrifuged and washed six times with PBS. The bacterial pellet was resuspended in 200 μL PBS. The pellet samples were loaded onto the SDS-PAGE and transferred onto a polyvinylidene fluoride (PVDF) membrane. The membrane was blocked with 5% skim milk and incubated with Mouse Anti-Trx-Tag mAb (ABclonal, Wuhan, Hubei, China). After washing three times with TBST, the membrane was incubated with HRP Goat Anti-Mouse IgG Antibody (ABclonal, Wuhan, Hubei, China). The proteins were visualized using the BeyoECL Plus Kit (Beyotime, Shanghai, China) following the manufacturer’s protocol. The experiment was performed in triplicate.

### 4.9. Bacterial Agglutination Experiment

The bacteria *V. parahaemolyticus* in their logarithmic growth phase were labeled with 0.1 mg/mL FITC. The FITC-labeled bacteria were diluted to 10^7^ cfu/mL and mixed with equal volume of 1 mg/mL rLvCrustin I-2 or rTrx. After incubation at room temperature for 1 h, the bacteria were observed under an optical Nikon TS100 microscope (Nikon, Tokyo, Japan). The experiment was performed in triplicate.

### 4.10. Intestinal Microbiome Analysis of LvCrustin I-2-Silenced Shrimp

Three groups including dsLvCrustin I-2 group, dsEGFP group and PBS group were devised in this experiment. A total of 45 shrimp were equally divided into these three groups and were injected with corresponding dsRNA and equal volume of PBS buffer, respectively, as described in section “Total viable bacteria count after dsRNA and *V. parahaemolyticus* injection”. At 48 h after injection, the intestines from five individuals were collected and pooled as one sample. Each group contained three replicate samples.

The microbial DNA of all the samples was extracted with the HiPure Stool DNA Kits (Magen, Guangzhou, Guangdong, China) following the protocol from the manufacturer. The PCR was performed to amplify the 16S rDNA V3-V4 region of the ribosomal RNA gene with primers 341F and 806R (Table S6). The PCR program was set as follows: 95 °C for 2 min, followed by 27 cycles at 98 °C for 10 s, 62 °C for 30 s, 68 °C for 30 s and a final extension at 68 °C for 10 min. The PCR fragments were purified with the AxyPrep DNA Gel Extraction Kit (Axygen Biosciences, Wujiang, Jiangsu, China) following the instructions from the manufacturer. The purified fragments were adjusted in equimolar and paired-end sequenced (2 × 250) on an Illumina platform according to the standard protocol.

The low-quality reads were removed with FASTP (version 0.18.0). The raw tags were generated and filtered with FLSAH (version 1.2.11) and QIIME (version 1.9.1), respectively. The chimeric sequences were checked via UCHIME algorithm (http://www.drive5.com/usearch/manual/uchime_algo.html, accessed on 16 June 2022). The effective tags were clustered into operational taxonomic units (OTUs) of ≥ 97% similarity with UPARSE pipeline. Taxonomies were assigned using a naive Bayesian model with RDP classifier based on SILVA Database (https://www.arb-silva.de/, accessed on 16 June 2022). The alpha diversity indexes and weighted unifrac distance matrix were calculated via QIIME. PCoA (Principal Co-ordinate Analysis) of weighted unifrac distances was calculated and plotted using R project. The biomarker features in different groups were screened with Metastats and LEfSe software. The functional prediction and phenotype classification were performed using BugBase.

### 4.11. Intestinal Transcriptome Analysis of LvCrustin I-2-Silenced Shrimp

A total of 30 shrimp were divided into two groups. The shrimp in *LvCrustin I*-2 silencing group were each injected with 8 μg *LvCrustin I*-2 dsRNA, and the shrimp in control group were injected with equal dose of EGFP dsRNA. At 48 h after dsRNA injection, the intestines from five individuals were collected and set as one sample. Six biological replicates were finally prepared for each group, designated as dsCru_1-6 and dsEGFP_1-6, respectively. The total RNA of these samples was extracted with RNAiso Plus reagent (TaKaRa, kusatsu, Shiga, Japan). RNA samples from dsCru_1-3 and dsEGFP_1-3 samples were used for transcriptome sequencing, and those from dsCru_4-6 and dsEGFP_4-6 samples were used to synthetize cDNA for qPCR analysis.

For Illumina sequencing, the mRNA was enriched using Oligo (dT) beads from the RNA samples and fragmented into short fragments and reverse transcribed into cDNA with random primers. The second-strand cDNA was synthesized and purified using QiaQuick PCR extraction kit (Qiagen, Venlo, The Netherlands). The short fragments were operated with end repair and the addition of poly (A). The cDNA library was constructed after the fragments were ligated with sequencing adapters and enriched by PCR amplification. The paired-end library was sequenced using Illumina HiSeq2500 by Gene Denovo Biotechnology Co. (Guangzhou, Guangdong, China).

The raw reads were filtered via FASTP (version 0.18.0) to obtain high-quality clean reads and mapped to ribosomal RNA (rRNA) database using Bowtie2 (version 2.2.8). Then the rRNA-mapped reads were removed. The paired-end clean reads were mapped to the reference genome (QCYY00000000) via HISAT2.2.4. The mapped reads of samples were assembled by reference-based approach using StringTie (version 1.3.1). To quantify the expression abundance of genes, the FPKM (fragment per kilobase of transcript per million mapped reads) value was calculated with StringTie software. The differential expression of genes between two different groups was performed using DESeq2 software. The genes with a false discovery rate (FDR) below 0.05 and absolute fold change above 2 were defined as differentially expressed genes (DEGs). The KEGG analysis was carried out with KEGG Automatic Annotation Server (http://www.genome.jp/tools/kaas/, accessed on 16 June 2022).

### 4.12. Elimination of Intestinal Microorganisms in Shrimp

The antibiotic-treated shrimp were obtained via reverse perfusion with antibiotic mixture (1 mg/mL ampicillin, 1 mg/mL neomycin, 1 mg/mL enrofloxacin, 1 mg/mL metronidazole and phenol red in PBS buffer) every 12 h for 48 h, and were cultured in sterile seawater at 25 °C. To verify the effectiveness of antibiotic treatment, the shrimp that were reverse perfused with equal volume of PBS buffer were set as controls. At 48 h after reverse perfusion, the intestines from three individuals were dissected and crushed in sterile PBS buffer as one sample, and the assay was performed in triplicate. The intestinal homogenate was diluted and seeded onto the TSB (LuQiao, Beijing, China) + 2% NaCl agar medium and cultured at 28 °C overnight, and then the total viable bacteria were counted.

A total of 30 antibiotic-treated shrimp with a body weight of 5.16 ± 1.04 g were divided into two groups: the dsLvCrustin I-2 group and dsEGFP group. The shrimp in the two groups were each injected with 8 µg corresponding dsRNA. The interference efficiency of *LvCrustin I*-2 dsRNA and the expression level of selected genes were detected via qRT-PCR analysis, as described in section “Total RNA Extraction and RT-qPCR Analysis”.

### 4.13. Statistical Analysis

The statistical significance between treatments and controls was analyzed using SPSS statistics 20 software by variance (ANOVA) and Duncan’s multiple comparisons. The significant differences were labeled with lowercase letters or stars at *p* < 0.05. In the microbiome analysis, the Alpha index comparison and BugBase analysis were calculated with *t*-test in R project. The PCoA analysis was calculated with ADONIS test. The significance was set at *p* < 0.05. The biomarker features in different groups at LDA score ≥ 2 were analyzed using LEfSe software.

## 5. Conclusions

**Figure 5:**
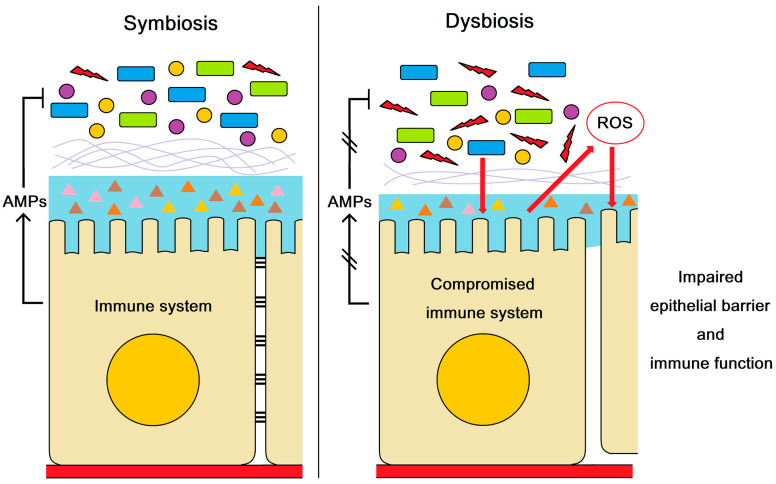
The role of AMPs in maintaining intestinal health and resisting pathogen infection. AMPs regulate intestinal microbiota balance. The compromised AMP expression resulted in dysbiosis of intestinal microbiota, which impaired the epithelial barrier and immune function in shrimp. The impairment of epithelial barrier and immune function may facilitate the *V. parahaemolyticus* invasion and the outbreak of AHPND.

According to the above evidence, we concluded that AMPs could maintain intestinal health and resist pathogen infection by regulating intestinal microbiota balance (Figure 5). The dysbiosis of intestinal microbiota induced excessive ROS production and impaired the epithelial barrier and immune function in shrimp, which facilitated the *V. parahaemolyticus* invasion. The present study revealed the molecular mechanism by which AMPs modulate the outbreak of AHPND in shrimp.

## Data Availability

All the raw data were deposited on the website http://dx.doi.org/10.12157/IOCAS.20220907.001 (accessed on 20 December 2022).

## Supplementary Materials

The following supporting information can be downloaded at: https://www.mdpi.com/article/10.3390/md21020130/s1, Figure S1: Nucleotide and amino acid sequence of LvCrustin I-2. The start codon and stop codon are boxed. The predicted signal peptide is waved underlined. The WAP domain is marked with dark background. The conserved cysteine residues in cysteine-rich region and WAP domain are underlined; Figure S2: Recombinant expression and microorganism-binding activity of LvCrustin I-2. SDS-PAGE of rLvCrustin I-2 produced in *E. coli* expression system. The expected band of rLvCrustin I-2 is indicated by an arrow. Lane M: protein ladder marker; Lane 1: total protein of *E. coli* before induction; Lane 2: total protein of *E. coli* after induction; Lane 3: inclusion of the induced *E. coli* lysate; Lane 4: supernatant of the induced *E. coli* lysate; Lane 5: purified rLvCrustin I-2. The microorganism-binding activity of rLvCrustin I-2 detected via Western blot employing an anti-Trx tag antibody. Recombinant rLvCrustin I-2 was sampled as the positive control; Figure S3: The agglutination activities of rLvCrustin I-2 to *V. parahaemolyticus.* rTrx was used as negative control; Figure S4: PCoA analysis based on weighted UniFrac distance of the intestinal microbiota in different samples. The *p*-value of ADONIS analysis was less than 0.05; Figure S5: The comparison of relative abundance of bacterial communities between control group and dsLvCrustin I-2 group. Ctrl 1-6, the microbiota samples from dsEGFP group and PBS group; dsCru 1-3, microbiota samples from LvCrustin I-2 knockdown shrimp. (A) Top ten abundant bacteria at the phylum level. (B) Top ten abundant bacteria at the class level. (C) Top ten abundant bacteria at the order level. (D) Top ten abundant bacteria at the family level. (E) Top ten abundant bacteria at the genus level; Figure S6: The score of linear discriminant analysis (LDA). The green and red colors indicated the increased and decreased bacteria in different taxa, respectively. The “Decreased” indicates the bacteria have higher proportion in control group than dsLvCrustin I-2 group; The “Increased” indicates the bacteria have higher proportion in dsLvCrustin I-2 group than control group. The length of the bar indicates the contributing degree of differences; Table S1: Minimal inhibitory concentration of rLvCrustin I-2 on pathogens; Table S2: Alpha diversity indexes of intestinal microbiota; Table S3: Summary of sequencing and assembly of the intestinal transcriptome from *L. vannamei*; Table S4: Expression levels of intestinal epithelial barrier related genes; Table S5: Expression levels of immune-related genes; Table S6: Nucleotide sequences of primers used in the present study.
