# Supplementary figures and images for: Crustin Defense against Vibrio parahaemolyticus Infection by Regulating Intestinal Microbial Balance in Litopenaeus vannamei
Source: Mar Drugs. 2023 Feb 17;21(2):130. doi: 10.3390/md21020130 (PMC9963704; doi:10.3390/md21020130)

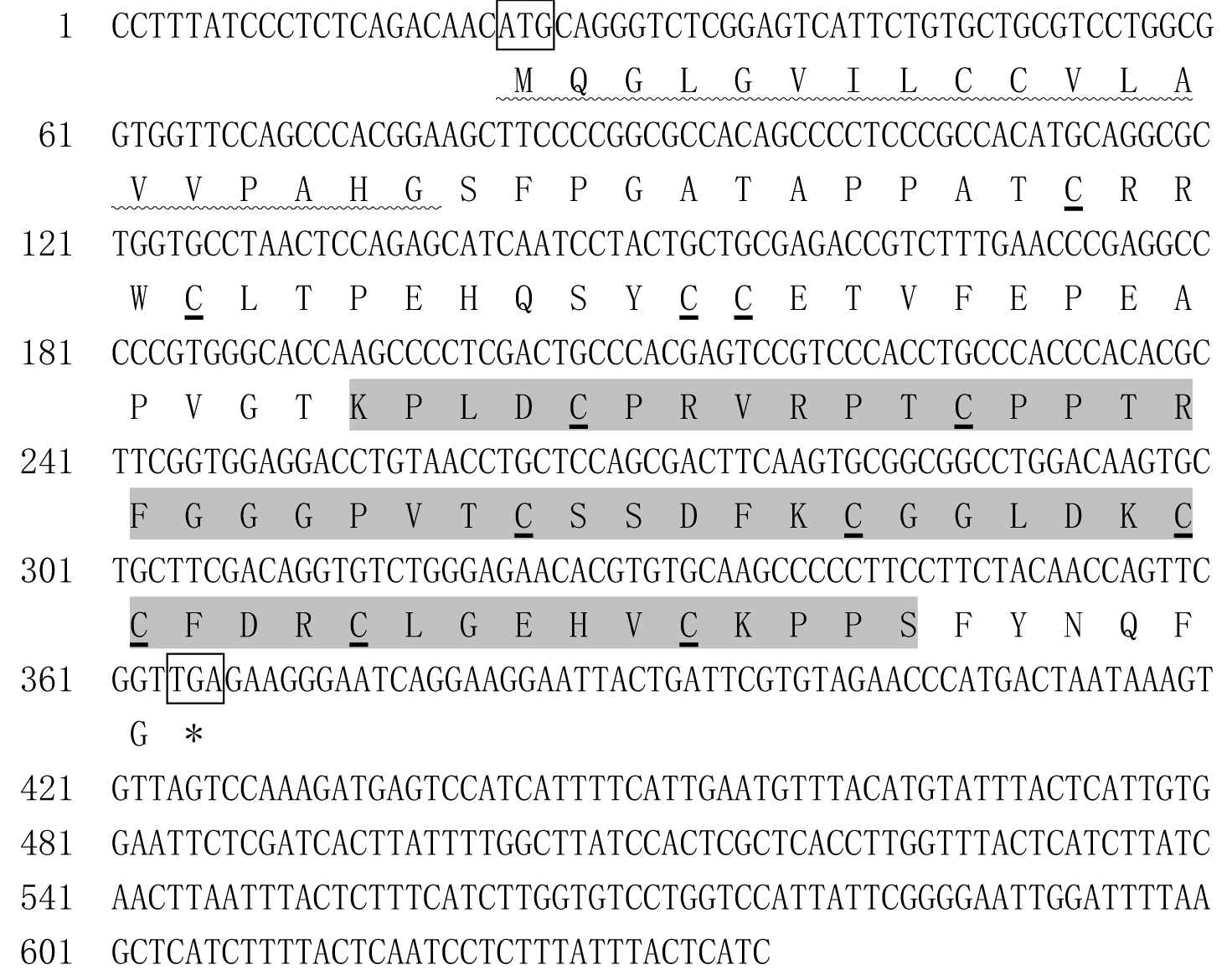

Supplement: Supplementary file 1 [file marinedrugs-21-00130-s001.zip › Figure S1.Nucleotide and amino acid sequence of LvCrustin I-2. The start codon and stop codon are boxed. The predicted signal peptide.tif]

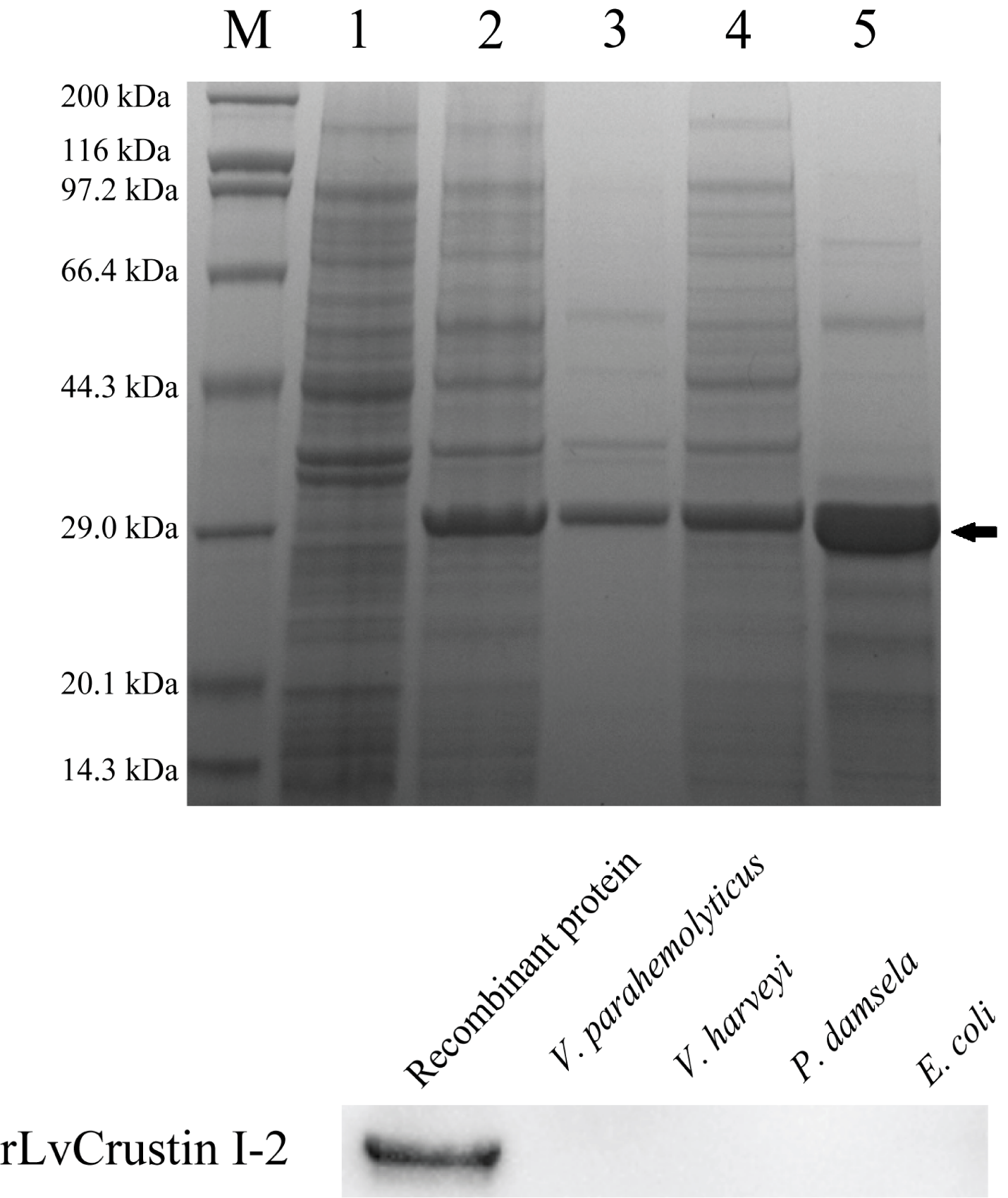

Supplement: Supplementary file 1 [file marinedrugs-21-00130-s001.zip › Figure S2.Recombinant expression and microorganism-binding activity of LvCrustin I-2. SDS-PAGE of rLvCrustin I-2 produced in E. coli .tif]

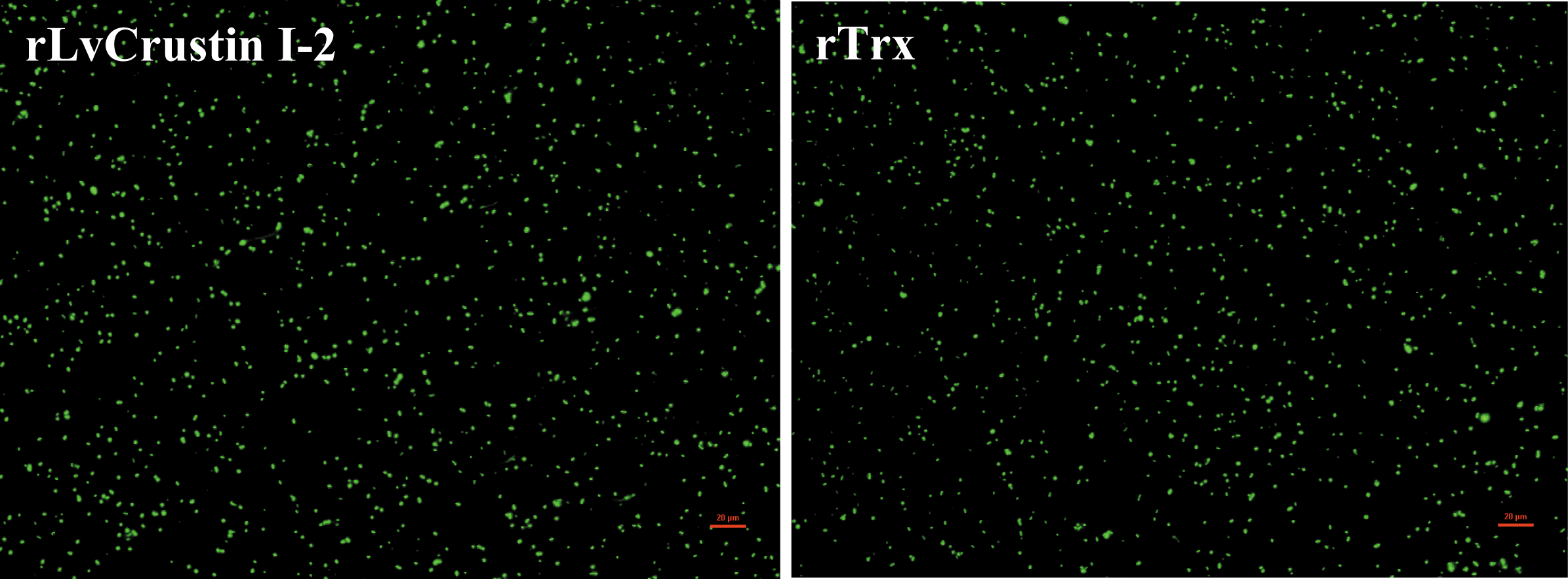

Supplement: Supplementary file 1 [file marinedrugs-21-00130-s001.zip › Figure S3.The agglutination activities of rLvCrustin I-2 to V. parahaemolyticus. rTrx was used as negative control.tif]

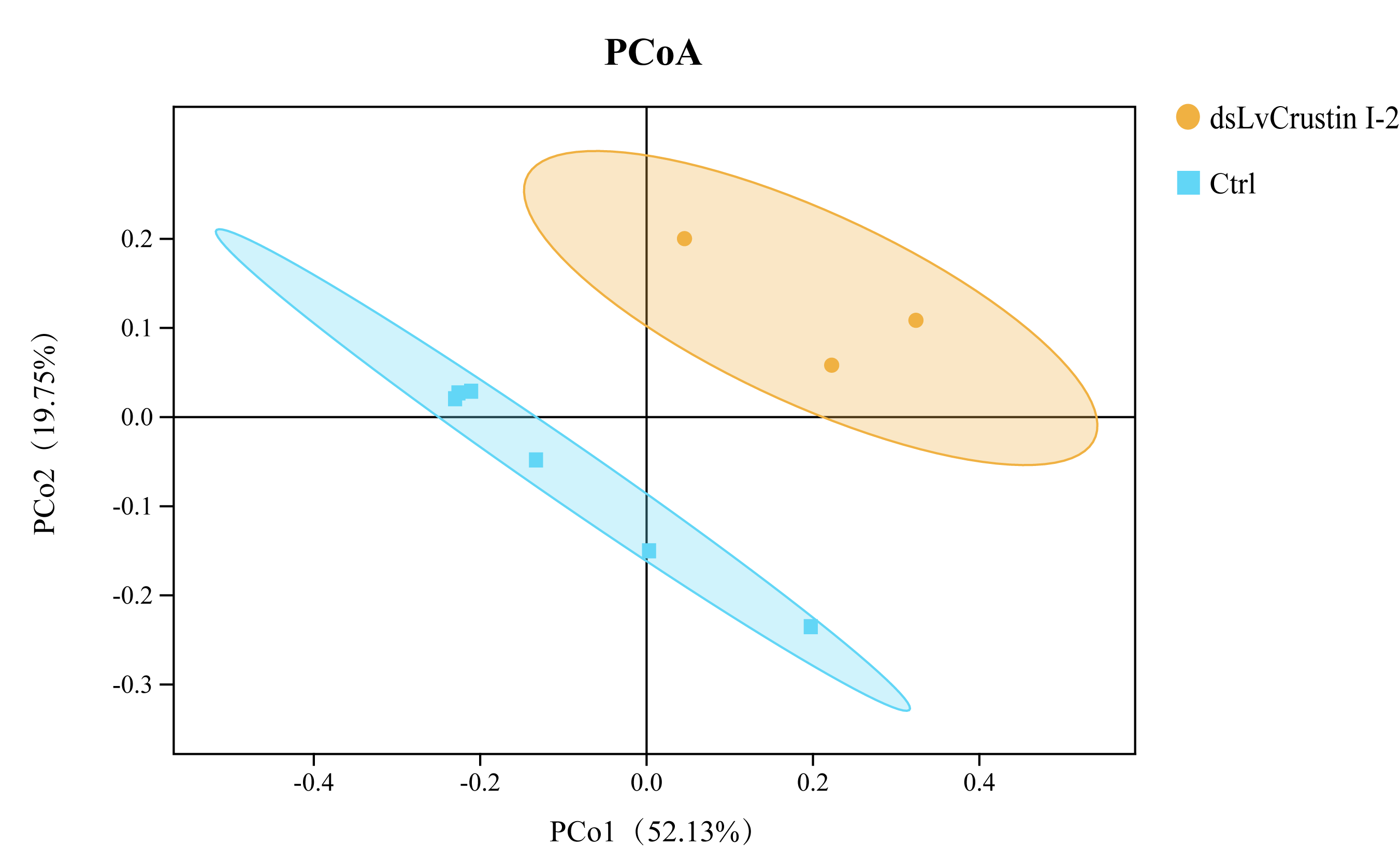

Supplement: Supplementary file 1 [file marinedrugs-21-00130-s001.zip › Figure S4.PCoA analysis based on weighted UniFrac distance of the intestinal microbiota in different samples. The p-value of ADONIS a.tif]

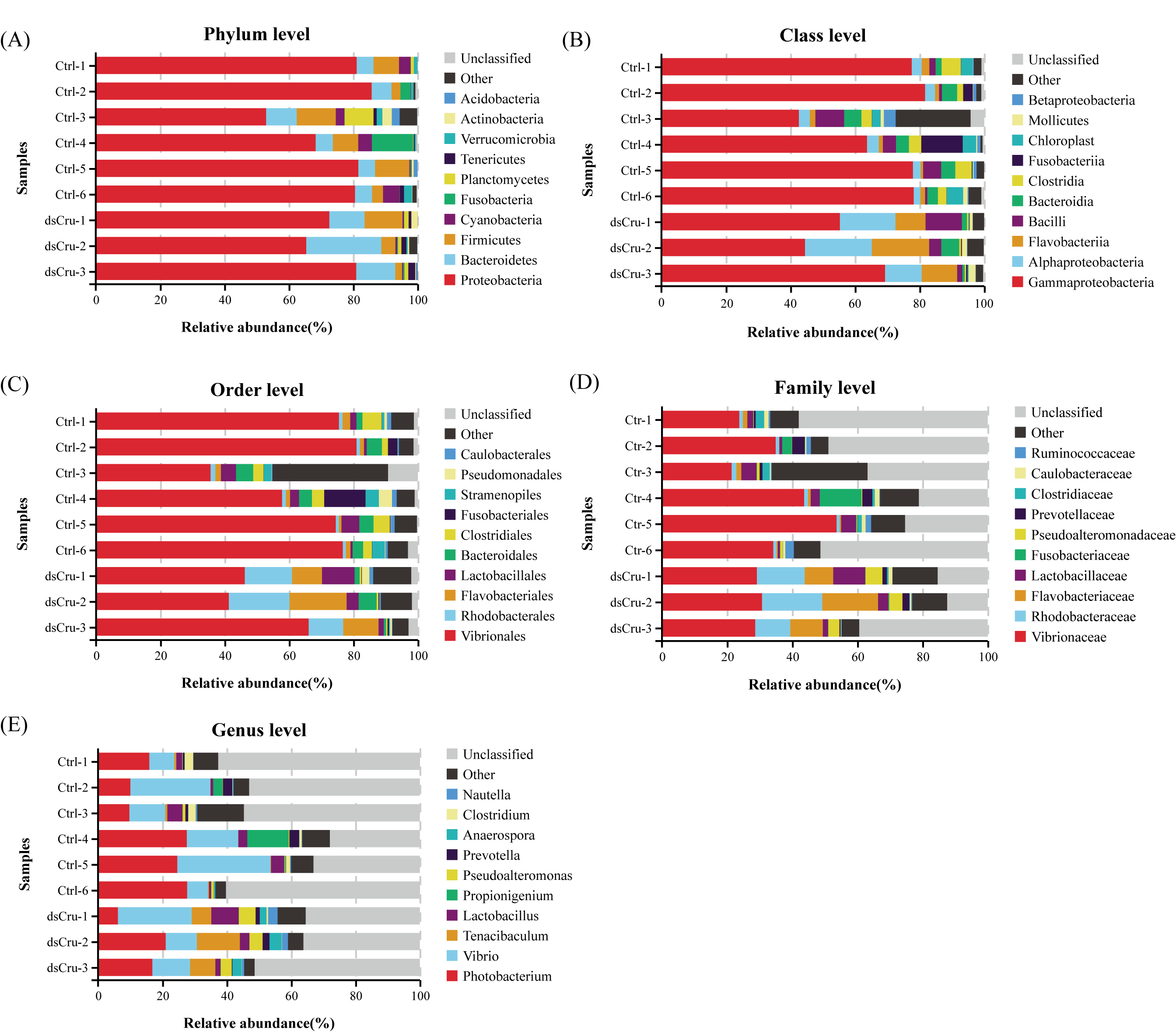

Supplement: Supplementary file 1 [file marinedrugs-21-00130-s001.zip › Figure S5.The comparison of relative abundance of bacterial communities between control group and dsLvCrustin I-2 group. Ctrl 1-6, th.tif]

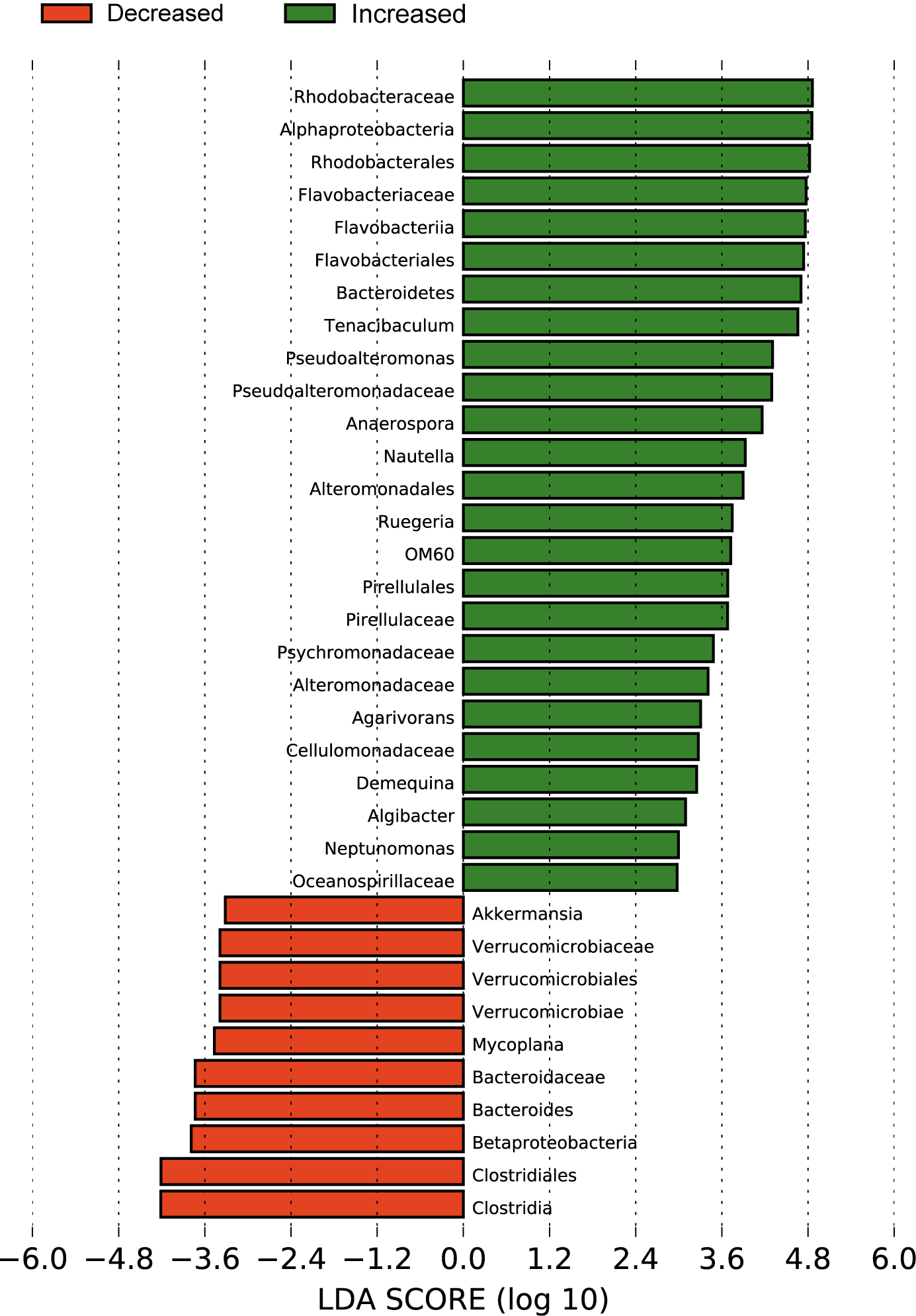

Supplement: Supplementary file 1 [file marinedrugs-21-00130-s001.zip › Figure S6.The score of linear discriminant analysis (LDA). The green and red colors indicated the increased and decreased bacteria in.tif]
